# Solubilization, purification, and ligand binding characterization of G protein-coupled receptor SMO in native membrane bilayer using styrene maleic acid copolymer

**DOI:** 10.7717/peerj.13381

**Published:** 2022-05-03

**Authors:** Lina Zhu, Hongxin Zhao, Yizhuo Wang, Chuandi Yu, Juanjuan Liu, Ling Li, Zehua Li, Jin Zhang, Han Dai, Junfeng Wang, Lei Zhu

**Affiliations:** 1 Institute of Physical Science and Information Technology, Anhui University, Hefei, China; 2 High Magnetic Field Laboratory, Key Laboratory of High Magnetic Field and Ion Beam Physical Biology, Hefei Institutes of Physical Science, Chinese Academy of Sciences, Hefei, China; 3 University of Science and Technology of China, Hefei, China; 4 School of Basic Medical Sciences, Nanchang University, Nanchang, China

**Keywords:** GPCR, Dissociation constant, Detergents, Styrene maleic acid copolymer nanodiscs, Smoothened receptors, NMR

## Abstract

Smoothened (SMO) protein is a member of the G protein-coupled receptor (GPCR) family that is involved in the Hedgehog (Hh) signaling pathway. It is a putative target for treating various cancers, including medulloblastoma and basal cell carcinoma (BCC). Characterizing membrane proteins such as SMO in their native state is highly beneficial for the development of effective pharmaceutical drugs, as their structures and functions are retained to the highest extent in this state. Therefore, although SMO protein is conventionally solubilized in detergent micelles, incorporating the protein in a lipid-based membrane mimic is still required. In this study, we used styrene maleic acid (SMA) copolymer that directly extracted membrane protein and surrounding lipids as well as formed the so-called polymer nanodiscs, to solubilize and purify the SMO transmembrane domain encapsulated by SMA-nanodiscs. The obtained SMA-nanodiscs showed high homogeneity and maintained the physiological activity of SMO protein, thereby enabling the measurement of the dissociation constant (K_d_) for SMO ligands SMO-ligands Shh Signaling Antagonist V (SANT-1) and Smoothened Agonist (SAG) using ligand-based solution nuclear magnetic resonance spectroscopy. This work paves the way for investigating the structure, function, and drug development of SMO proteins in a native-like lipid environment.

## Introduction

G protein-coupled receptors (GPCRs) constitute the largest membrane protein family and are essential, but elusive, drug targets in several diseases. As a member of the class F GPCR family, the smoothened (SMO) protein is regulated by the Hedgehog (Hh) signal that can be transmitted to PKA, CK1α, GSK3β, and other downstream signaling molecules. These signaling molecules can activate glioma related transcription factor Gli and promote cell proliferation (Briscoe & Therond, 2013). SMO is an attractive target for congenital disabilities and various cancers, including medulloblastoma and basal cell carcinoma (Zhang et al., 2018). Structural biology studies have shown that the deep seven-transmembrane (7TM) pocket of SMO is the main binding site for many natural and synthesized small-molecule drugs (Qi et al., 2020; Wang et al., 2014; Wang et al., 2013). Antagonists such as cyclopramide, vismodegib, sonidegib, SMO-ligands Shh Signaling Antagonist V (SANT-1), and Smoothened Agonist (SAG) and its derivatives, have been successfully developed using cell-based assays (Sekulic & Von Hoff, 2016; Zhang et al., 2018).

As a vital class of membrane proteins, GPCRs such as SMO requires a lipid bilayer environment for their proper folding and cellular localization. To characterize SMO protein *in vitro* using various biochemical and biophysical techniques, the protein must be extracted from the membrane and reconstituted into a suitable membrane mimic, thereby maintaining their structural and functional integrity (Qi et al., 2020; Wang et al., 2014; Wang et al., 2013). Detergent micelles have achieved considerable success and are conventionally used for membrane proteins solubilization. However, to avoid the loss of function due to improper detergents, extensive benchwork is normally required to optimize the detergents and their assembly conditions.

As an alternative, nanodiscs provide a more protein-friendly and generally applicable system that ensures a native-like lipid bilayer environment. The first developed nanodisc system was a disc-shaped nanoparticle formed by wrapping the lipid bilayer core with two membrane scaffold protein (MSPs) molecules in a belt-like configuration (Bayburt, Carlson & Sligar, 1998; Bayburt, Grinkova & Sligar, 2002). This system has been successfully used in structural biology, physiological function analysis, and drug screening for several membrane proteins, such as GPCRs (Lavington & Watts, 2020; Zhang et al., 2021), photosynthetic reaction center (Crisafi & Pandit, 2017; Lu et al., 2016), cytochrome oxidases (Denisov & Sligar, 2011), and transporters (Autzen et al., 2018; Gao et al., 2016; Li, Rietmeijer & Brohawn, 2020). In addition to MSP, other scaffolds such as saposine-A lipoprotein (Frauenfeld et al., 2016) and amphiphilic peptides (Carlson et al., 2018; Kariyazono et al., 2016; Larsen et al., 2016; Zhang et al., 2016) have also exhibited efficient nanodisc assembly. Although the protein- and peptide-based nanodiscs have achieved successes in a number of applications, there remain some obstacles to their efficient use. For instance, detergent is invariably required in the solubilization procedure but often causes irreversible protein denaturation. Additionally, the presence of scaffold proteins or peptides interferes with the use of various protein spectroscopy techniques in sample characterization.

Except for proteins and small peptides, amphiphilic polymers have also been developed to build a new nanodisc platform. The first and the most widely platform uses styrene maleic acid copolymer (SMA)—an amphiphilic polymer synthesized by polymerizing hydrophobic styrene and hydrophilic maleic anhydride monomers in different proportions (Knowles et al., 2009). The size of SMA-based nanodisc can be adjusted by changing the ratio of polymer to lipid, in the range of 10 to 30 nm diameter (Craig et al., 2016; Zhang et al., 2015; Ravula et al., 2018b). In addition, SMA copolymer derivatives were also developed to expand the applications of polymer nanodiscs. For example, a small molecular weight copolymer SMA–ethanolamine (SMA-EA) increased the maximum nanodisc size limit to approximately 60 nm in diameter (Ravula et al., 2017b). SMA–ethylene diamine (SMA-ED) and styrene maleimide–amine (SMA-dA) polymers exhibited different levels of pH-dependent stability and enhanced tolerance towards divalent metal ions (Ravula et al., 2017a). Styrene maleimide–quaternary ammonium (SMA-QA), prepared using the pH-independent and non-chelating quaternary ammonium group, showed high stability under wide ranges of pH and divalent metal ion concentration (Ravula et al., 2018b).

Large SMA-nanodiscs are well aligned in magnetic fields and are suited for solid-state nuclear magnetic resonance (NMR) studies (Radoicic, Park & Opella, 2018; Park et al., 2020). Small size SMA-nanodiscs can also be used for solution NMR studies. ^15^N-labeled cytochrome-b5 (Cyt-b5) were reconstituted into SMA-QA nanodiscs, and its well dispersed 2D ^1^H/^15^N transverse relaxation optimized spectroscopy-heteronuclear single quantum coherence (TROSY-HSQC) NMR spectra were obtained successfully (Ravula et al., 2017b; Ravula et al., 2018a).

A significant advantage for membrane protein solubilization is that SMA can directly extract membrane proteins encapsulated by natural lipids from biological membranes to form disc-like nanoparticles while maintaining the protein’s structure and function. Compared with conventional detergent-based extraction, the detergent-free extraction of membrane proteins using SMA improves their solubility and stability. Thus this process has been used in studies on membrane proteins such as potassium channel KcsA (Dorr et al., 2014) and ROMK (Krajewska & Koprowski, 2021), Human Multidrug Resistance Protein 4 (MRP4/ABCC4) (Hardy et al., 2019), lipopolysaccharide-transporter complex LptB2FG (Baeta et al., 2021), and GPCRs (Bada Juarez et al., 2020; Jamshad et al., 2015; Juarez et al., 2020).

**Figure 1:**
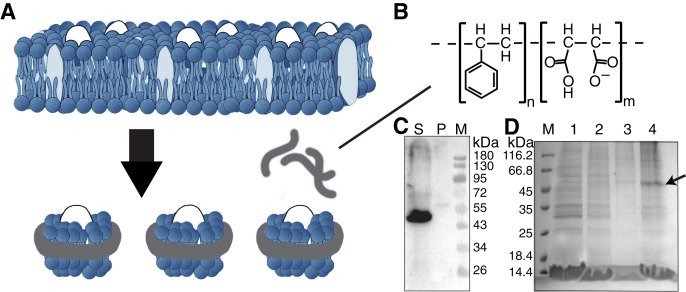
Solubilization and purification of SMO transmembrane domain using SMA copolymers. (A) Schematic representation of membrane protein extraction and nanodisc formation by SMA. (B) Chemical structure of the SMA copolymer. (C) Western blotting of SMO solubilized directly from cells by SMA using an anti His-Tag antibody. The supernatant and pellet after solubilization were separated through ultracentrifugation. S, supernatant; P, pellet; M, protein marker. (D) SDS-PAGE results showing the nickel affinity chromatography-purified SMO encapsulated in SMA-nanodiscs. The arrow indicates the SMO band. M: protein marker; Lane 1: flow through; Lane 2: wash with 25 mM imidazole; Lane 3: wash with 50 mM imidazole; Lane 4: elution with 250 mM imidazole.

Although SMO protein has been successfully reconstituted in a few detergent systems (Qi et al., 2020; Wang et al., 2014; Wang et al., 2013), the ratio of nonideal mimetic of detergents to the lipid membrane environment still limits their potential applications. In this study, we used the SMA copolymer to directly solubilize and purify the transmembrane regions of SMO protein (190–555 amino acids) from Sf9 insect cells using a detergent-free method (Figs. 1A and 1B). Solution NMR study verified the physiological activity of SMO by determining the dissociation constant (K_d_) with an antagonist SANT-1 and an agonist SAG. GPCR proteins, such as SMO, embedded in SMA-nanodiscs may be used in ligand-based NMR to characterize the ligands-binding property of SMO, and provide insights into the protein’s structure, function, and potential in drug discovery.

## Materials and Methods

### SMO proteins expression

The gene encoding the human SMO transmembrane domain (residues 190-555) fused with N-terminal 10-His tag and thermostabilized apocytochrome b562RIL (Chun et al., 2012) was introduced into the pFastBac1 vector through EcoRI and HindIII restriction sites. The recombinant protein was expressed in Sf9 insect cells (purchased from ThermoFisher, Waltham, MA, USA) using the Bac-to-Bac Baculovirus System (ThermoFisher, Waltham, MA, USA) for 48 h after infecting thrice. Western blotting was performed to detect the expression of the recombinant protein. The cells were harvested by centrifuging at 3,000 rpm for 10 min at 4 °C. The obtained wet cell pellet typically weighed approximately 2 g per 500 ml of the culture.

### Solubilization and purification of SMO using SMA copolymer

SMA (Sigma-Aldrich, SAE0062, Styrene:Maleic Anhydride Copolymer 2:1, Pre-hydrolyzed) was dissolved in a 50 mM Tris buffer (pH 8.0) for a final concentration of 6%. The cell pellet was resuspended in a 50 mM Tris buffer (600 mM NaCl and 30 mM KCl, pH 8.0) to obtain a concentration of 0.2 g/ml and incubated with 5 μg/ml DNase on ice for 45 min. The suspension was mixed with the SMA solution at a volume ratio of 1:1 and then subjected to ultrasonication (30 s burst, on ice) before overnight gentle stirring at room temperature. The membrane component encapsulated by SMA was collected *via* ultracentrifugation at 100,000 g for 45 min. Because the charge interaction between protein and polymer may affect the solubilization process (Ravula et al., 2018a), we tested the yield of negatively charged SMO (pI 6.469, −10.262 net charge at pH 8.0) by western blotting with an anti-His-Tag antibody (1:1,000; #2365; Cell Signaling Technology, Danvers, MA, USA). The supernatant was incubated with Ni-NTA resin overnight at 4 °C. The protein was eluted sequentially with 20 mM, 50 mM, and 250 mM imidazole and visualized *via* SDS-PAGE analysis. The purified SMO protein in SMA-nanodiscs was further purified and analyzed *via* gel filtration chromatography in a buffer containing 50 mM Tris, 300 mM NaCl, and 15 mM KCl (pH 8.0) and the Superdex 200 10/300 GL column (Cytiva, Marlborough, MA, USA) with a UNION protein purifier (Union-Biotech, Shanghai, China). The obtained SMO protein was probed using western blotting with an anti-His-Tag antibody, and the protein concentration was determined using the BCA assay (#23227; Thermo Fisher Scientific, Waltham, MA, USA). The final yield of SMO protein was approximately 100 μg in a single extraction and purification experiment.

For SMA-nanodiscs without SMO, uninfected Sf9 cells were used for assembly. The entire method was the same except that the Ni-NTA purification step was skipped.

### Dynamic light scattering

Dynamic light scattering (DLS) experiments were performed on a Malvern Zetasizer Nano ZS equipped with a He-Ne laser as the light source (wavelength: 633 nm; scattering angle: 173°) at 25 °C. The sample was diluted with a buffer containing 50 mM Tris, 300 mM NaCl and 15 mM KCl (pH 8.0; final concentration, 20 μg/ml), and one milliliter of each sample was measured (11 runs of 10 s) in triplicate in a single-use disposable cuvette (Malvern, DTS0012) with a 10-mm path length. Data was analyzed using the Zetasizer Software 7.11 (Malvern Instruments Ltd., Malvern, UK).

### Transmission electron microscopy imaging

For transmission electron microscopy (TEM), 2.5 μl sample solution (10 μg/ml protein) was placed on Carbon-coated copper grids (Zhongke Microscope). The grids were then stained with 2% uranyl acetate solution for 1 min, and the excess staining solution was removed by blotting with filter paper. Air-dried samples were stored in a desiccator until observation using TEM. The TEM experiments were performed using the FEI Tecnai 12 TEM (120 kV). The size distribution of the nanoparticles was analyzed using the ImageJ software.

### NMR titration

A 0.5 mM SANT-1 (aladdin, S129830) was dissolved in D_2_O buffer containing 10 mM Tris, 300 mM NaCl, 15 mM KCl and 10% deuterated DMSO at pH 8.0. On the other hand, 0.5 mM SAG (SML1314; Sigma-Aldrich, St. Louis, MI, USA) was dissolved in the same buffer excluding DMSO. The purified SMO proteins in SMA-nanodiscs were sequentially titrated into substrate samples at the indicated concentration. The ^1^H spectra of each titration were obtained in triplicate using the Bruker AVANCE III 850 or 600 MHz spectrometers equipped with cryogenic probes at 298 K. The peak heights were of 7.97 ppm and 2.54 ppm for SANT-1 and SAG, respectively, which were normalized relative to the 2,2-Dimethyl-2-silapentane-5-sulfonate (DSS) signal using the Bruker Topspin 4.0 software. The dissociation constant was determined by fitting the binding curve with the one-site binding model.

## Results

The baculovirus/insect expression system has been extensively used to overexpress a lot of membrane proteins such as GPCRs for crystallization and cryo-electron microscopy studies (Qi et al., 2020; Wang et al., 2014; Wang et al., 2013). The 7TM helical domain of SMO (190–555) fused with a common GPCR partner, thermostabilised apocytochrome b562RIL (BRIL) was expressed in Sf9 insect cells. A 10-His tag was introduced to the N-terminus of the recombinant protein to enable easy purification *via* the immobilized metal affinity chromatography.

**Figure 2:**
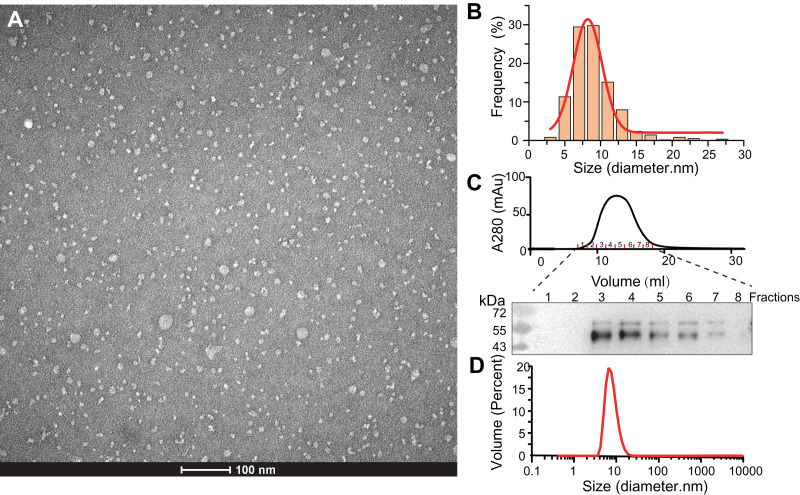
Characterization of SMO SMA-nanodiscs. (A) TEM microimage of uranyl acetate stained SMO SMA-nanodiscs. (B) Particle size distribution in the TEM image. The average diameters of the particle were fitted to 8.2 ± 2.4 nm. (C) Gel filtration chromatography of SMO SMA-nanodiscs. The fractions were analyzed by western blotting using an anti His-Tag antibody. (D) DLS measurements of SMO SMA-nanodiscs. The average diameter of the particles was determined to 7.7 ± 2.5 nm.

For SMO solubilization, 3% w/v of the SMA copolymer with a 2:1 ratio of styrene to maleic acid was directly added into the buffer-resuspended cell pellet to extract the membrane component and form SMA-nanodiscs to obtain the overexpressed SMO protein surrounded by native lipids that may contribute to the protein’s structure and function. When the pellet was visibly clear after ultrasonication, the nonsolubilized material was removed by centrifuging at 100,000 g for 45 min at 4 °C. Of the SMO protein, >90% was successfully solubilized using SMA, as indicated by the results of western blotting using an anti-His antibody (Fig. 1C). The resulting solution was purified using nickel-affinity chromatography. The result of SDS-PAGE showed that SMO was eluted as a single (nearly 50 kDa) band in fractions containing 250 mM imidazole, which is consistent with the result of western blotting (Fig. 1D). Afterward, gel filtration chromatography was performed using SMO SMA-nanodiscs, which revealed a broad peak ranging from 7 to 20 ml; the SMO protein was mainly distributed in fractions 3–5, as evident from the results of western blotting (Fig. 2C). This result indicated that there were still many empty nanodiscs even after Ni-NTA purification. Finally, we used a mixture of only fractions three and four for the subsequent experiments.

The purified SMA-nanodiscs were analyzed using TEM and DLS to further characterize their particle size and homogeneity. The TEM images of the negatively stained sample showed dispersed disks with average particle diameters of 8.2 ± 2.4 nm (Figs. 2A and 2B), which is consistent with the diameter of 7.7 ± 2.5 nm derived from DLS experiments (Fig. 2D) (negative staining often overestimates particle sizes; Bayburt & Sligar, 2003; Knowles et al., 2009). Because the 7-TM domain of SMO proteins are nearly 30 Å in diameter, one nanodisc typically contains only one protein, which is in line with the findings related to its monomeric state reported in many studies (Qi et al., 2020; Wang et al., 2014; Wang et al., 2013). SMO SMA-nanodiscs comprised approximately 10 phosphatidylcholine (PC) molecules per SMO protein as evident from the results of the the phosphate assay and BCA assay for the determination of PC and protein concentrations, respectively; this finding supports that of previous studies (Knowles et al., 2009; Gulati et al., 2014).

NMR spectroscopy is a versatile technique that is used to measure protein’s ligand-binding affinity and can identify new candidates for drug discovery (Hanzawa & Takizawa, 2010; Shimada et al., 2019). To verify the physical activity of SMO incorporated in SMA-nanodiscs and explore its potential in the application of NMR-based drug screening, 1D ^1^H NMR spectra were used to compare the signal-intensity changes of ligands in the bound state relative to the free-state, as the specific binding of SMO to the ligand causes line broadening because of the increase in transverse relaxation rates (R2). We characterized the binding profiles of two commercially available ligands, SANT-1 and SAG to SMO.

**Figure 3:**
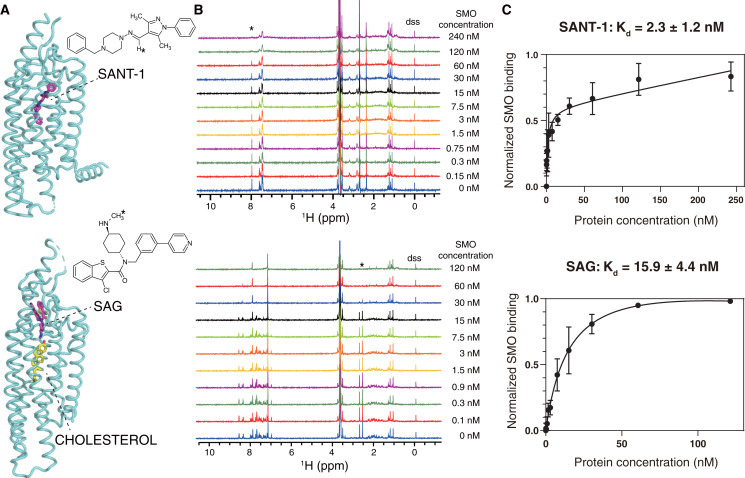
NMR titrations for the K_d_ determinations of SANT-1 (top) and SAG (bottom) to SMO incorporated in SMA-nanodiscs. (A) Binding models of SANT-1 and SAG to SMO transmembrane domain (from crystal structure, PDB ID: 4n4w and 6xbl, respectively). SMO is shown in cartoon representation, ligands are shown in stick representation. Asterisks indicate the protons whose resonances are used for K_d_ determination. (B) ^1^H spectra for ligands titrated with SMO incorporated in SMA-nanodiscs. Asterisks indicate the peaks used for K_d_ determination. (C) Binding curves fitting for SANT-1 and SAG during their interaction with SMO incorporated in SMA-nanodiscs. A one-site binding model was used to fit the curve. Error bars show the standard deviation (SD) of three independent experiments.

SANT-1 is a potential antagonist that binds to the deep cavity formed by the 7TM helical bundle of SMO, with a strong binding affinity (K_d_ = 1.2 nM; Fig. 3A, top panel) (Chen et al., 2002; Wang et al., 2014). We titrated a series of SMO SMA-nanodiscs into SANT-1 and observed that the proton signal of SANT-1 significantly broadened along with the increase of protein concentration, indicating a specific binding process (Fig. 3B, top panel). The K_d_ value was then fitted to 2.3 ± 1.2 nM based on the relative peak heights of the only imine CH proton that led to a single and separated resonance at 7.97 ppm (Fig. 3C, top panel).

SAG is an agonist that can block an endogenous sterol in the 7TM bundle and facilitate the change to the active conformation of SMO to recruit heterotrimeric G_i_ (K_d_ = 59 nM, Fig. 3A, bottom panel) (Chen et al., 2002; Qi et al., 2019; Qi et al., 2020). Using the same titration protocol used for SANT-1, the K_d_ value for the interaction of SMO and SAG was determined to be 15.9 ± 4.4 nM using the relative peak heights of the CH3 protons of the methylamino group at 2.54 ppm (Figs. 3B and 3C, bottom panel).

The slight discrepancy in the K_d_ value might have resulted from the differences between the *in vitro* NMR-based method and previously reported cell-based methods as well as a possible qualification error of protein concentrations. As a negative control, SMA-nanodiscs without SMO were also added into the ligand samples, but no significant binding was observed (Fig. S1). Thus, SMO protein extracted and purified using SMA may perfectly retains its native activity, and NMR spectroscopy is the ideal method used to measure the binding affinity of SMO with its ligand.

## Discussion

GPCRs are integral membrane proteins that require environments containing an aqueous surface and a hydrophobic interior that mimic the cell membrane and contribute to the overall functional protein folding of the receptor. Although studying GPCRs *in situ* is not problematic, the structural and biophysical studies often require the protein to be extracted from the cell membrane. This process usually require the incubation of detergent micelles to remove the native membrane, followed by substantial purification and reconstitution into a proper membrane mimic. However, disruption of the lipid bilayer by detergent may cause irreversible denaturation or strip away some special lipids or other components that affect the protein’s conformation and function (Wheatley et al., 2016). As an alternative agent to detergent for the solubilization of membrane proteins, SMA incorporates into the membrane and forms nanoscale lipid particles using a native membrane bilayer, thus preserving the natural state of the membrane protein to the highest possible extent (Wheatley et al., 2016).

In this study, we successfully extracted SMO encapsulated in the native lipid bilayer without using detergents. Compared with the detergent purified SMO proteins, there are several advantages of the approach that does not involve detergent use. (i) SMA use is a simple but effective way to extract membrane protein while avoiding detergents and screening optimum conditions for solubilization. To the best of our knowledge, this study is the first to report that active SMO can be assembled into nanodiscs. The only optimization performed in this study was the screening of the optimum SMA concentration and its ratio to the cells. (ii) SMO SMA-nanodiscs retain the native composition of lipids associated with the receptor, which is essential for maintaining the conformation and function of membrane proteins. (iii) Purified SMO SMA-nanodiscs have high homogeneity and can be used directly for biophysical characterization.

NMR-based ligand screening is an invaluable tool in the drug discovery industry. Various 1D NMR methods for detecting ligands that probe various NMR parameters (*e.g*., chemical shift changes, relaxation times, diffusion rates, saturation transfer differences [STD], and transfer NOEs), enable timely and highly desirable high-throughput NMR screening for the discovery of new potential ligands (Hanzawa & Takizawa, 2010; Shimada et al., 2019). Although associated with several challenges, ligand-based NMR methods have been successfully used in studies on GPCR-ligand complex structures as well as therapy drug screening; the receptors studied include GPR40 (Bartoschek et al., 2010), κ-opioid receptor (O’Connor et al., 2015), chemokine receptor CXCR1 (Berkamp et al., 2017; Park et al., 2017), and bradykinin receptors (Joedicke et al., 2018). In the present study, we demonstrated that SMA-nanodiscs can measure SMO’s ligand binding affinity by monitoring the signal changes of low-molecular-weight ligands. This system can be further applied in high-throughput drug screening using line-broadening change and STD NMR experiments.

## Conclusions

To the best of our knowledge, this study is the first to report the purification of the GPCR SMO by encapsulating it directly from a membrane into SMA-nanodiscs, without any exposure to detergents. NMR methods enables the *in vitro* characterization of SMO’s binding with two different ligands. Furthermore, they have potential applications in drug discovery including screening small-molecule ligands as well as specific antibodies of SMO within a lipid environment.

## Supplemental Information

10.7717/peerj.13381/supp-1 Supplemental Information 1 ^1^H spectra for SMA-nanodiscs without SMO titrated into 0.5mM SANT-1 (A) and SAG (B). SMA-nanodiscs were prepared using uninfected Sf9 cells. The concentration of SMA-nanodiscs added was equal to that of the 1 μM SMO SMA-nanodiscs, as determined by measuring the absorbance at 280 nm. # indicates a broadened peak at around 6.8–7.3 ppm corresponding to the aromatic protons of SMA.

10.7717/peerj.13381/supp-2 Supplemental Information 2 Raw data for Figure 1C.

10.7717/peerj.13381/supp-3 Supplemental Information 3 Raw data for Figure 1D.

10.7717/peerj.13381/supp-4 Supplemental Information 4 Raw data for Figure 2A.

10.7717/peerj.13381/supp-5 Supplemental Information 5 The raw curve data of gel filtration experiment for SMO SMA-nanodiscs shown in Figure 2C.

10.7717/peerj.13381/supp-6 Supplemental Information 6 The full-view western blotting image shown in Figure 2C visualizing the result of gel filtration experiment for SMO SMA-nanodiscs.

10.7717/peerj.13381/supp-7 Supplemental Information 7 Raw data for Figure 2D.

10.7717/peerj.13381/supp-8 Supplemental Information 8 Raw data for Figure 3B, SAG.

10.7717/peerj.13381/supp-9 Supplemental Information 9 Raw data for Figure 3B, SANT-1.

10.7717/peerj.13381/supp-10 Supplemental Information 10 Raw data for Figure 3C.

## Additional Information and Declarations

### Data Availability

The following information was supplied regarding data availability: The raw data are available in the Supplemental Files.
