# Supplementary figures and images for: Solubilization, purification, and ligand binding characterization of G protein-coupled receptor SMO in native membrane bilayer using styrene maleic acid copolymer
Source: PeerJ. 2022 May 3;10:e13381. doi: 10.7717/peerj.13381 (PMC9074879; doi:10.7717/peerj.13381)

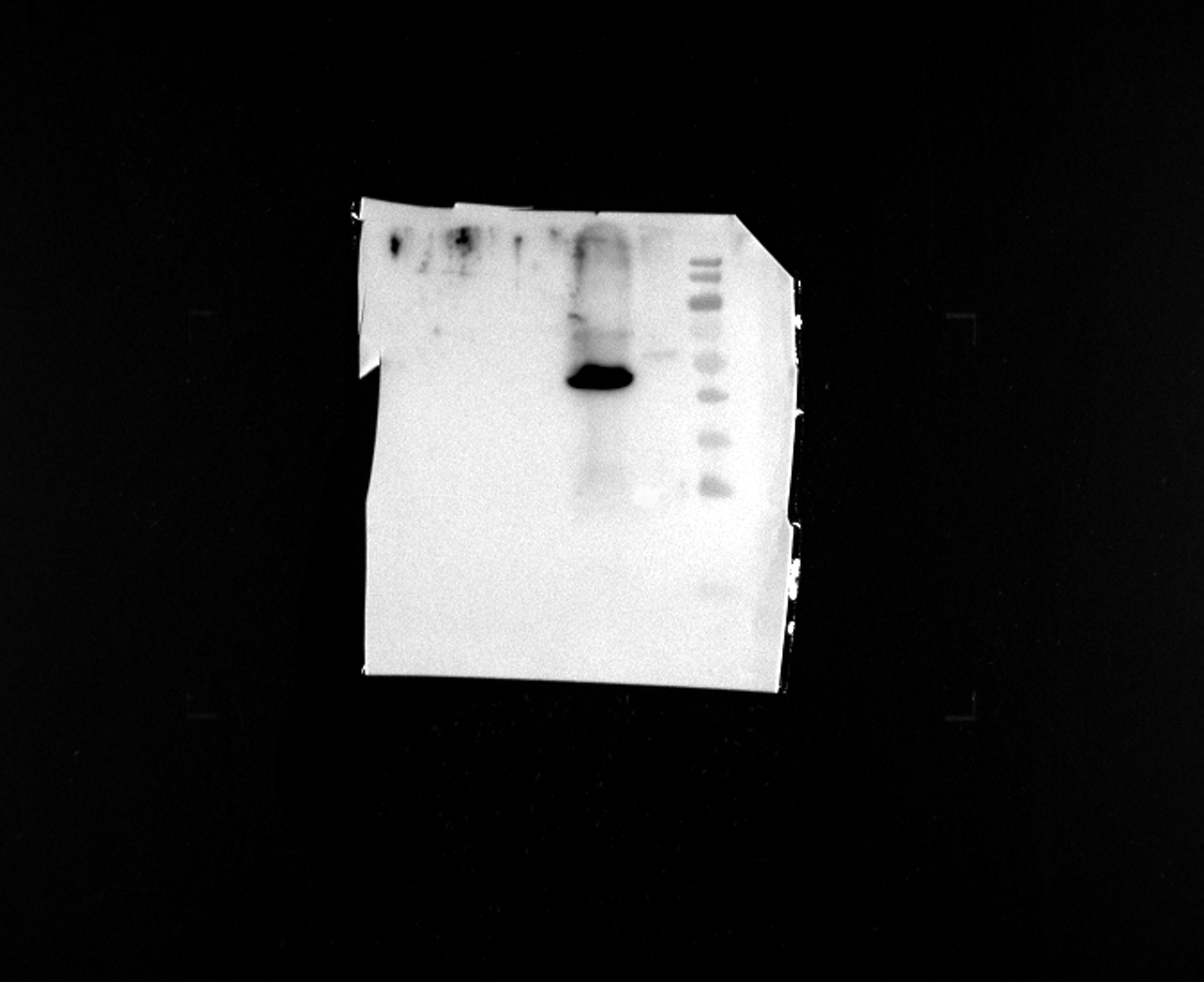

Supplement: Supplemental Information 2 [file peerj-10-13381-s002.tif]

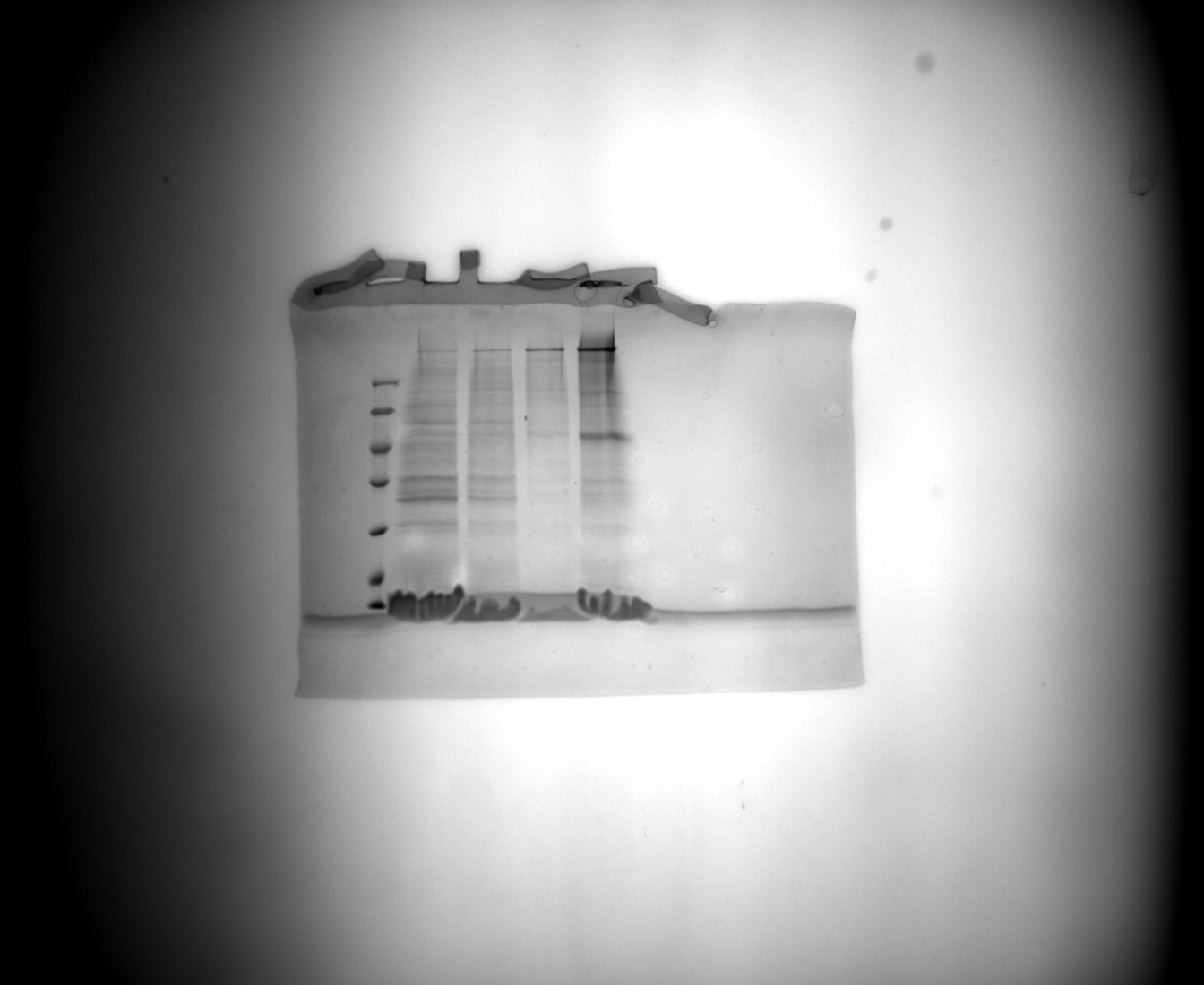

Supplement: Supplemental Information 3 [file peerj-10-13381-s003.tif]

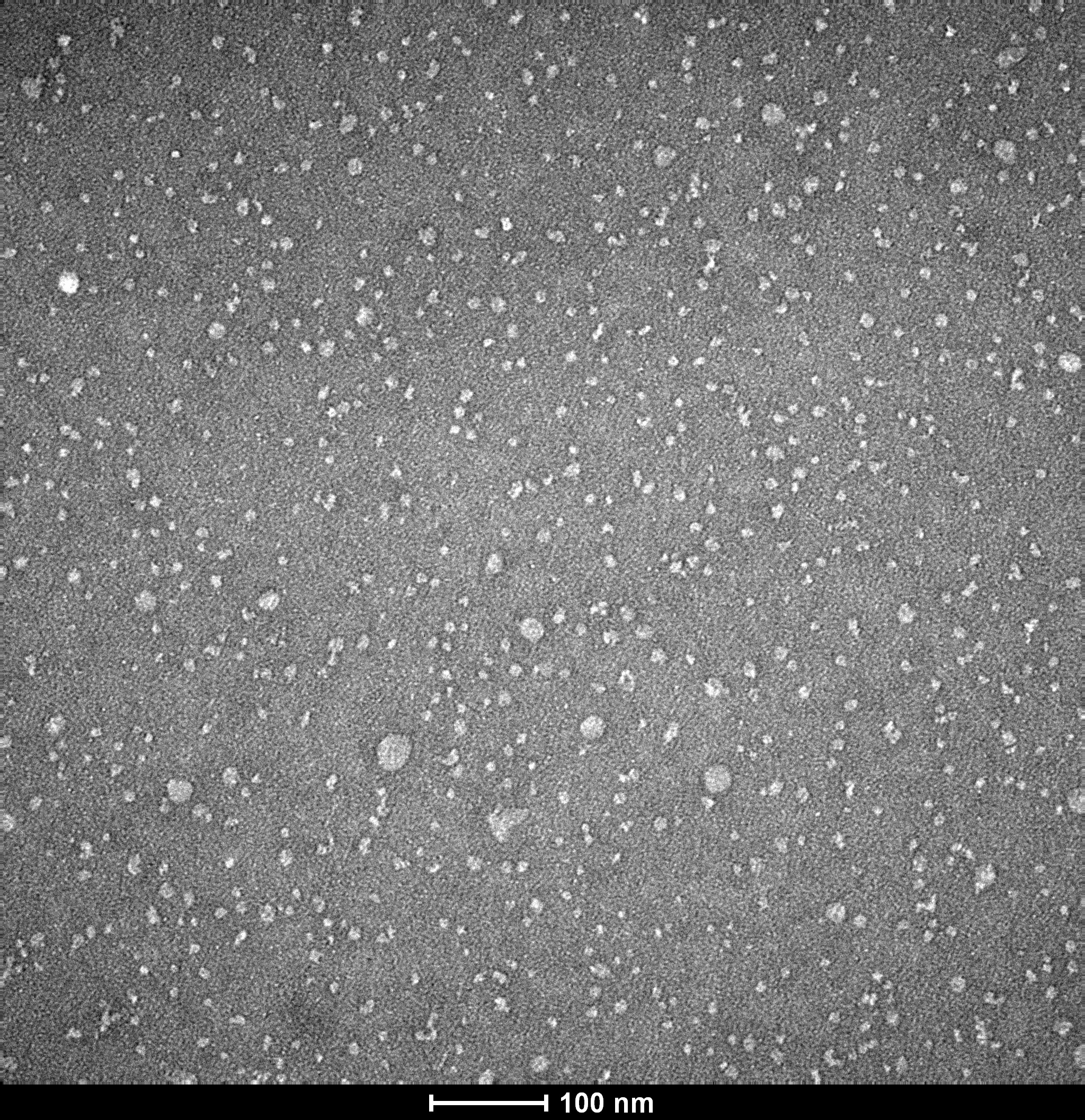

Supplement: Supplemental Information 4 [file peerj-10-13381-s004.jpg]

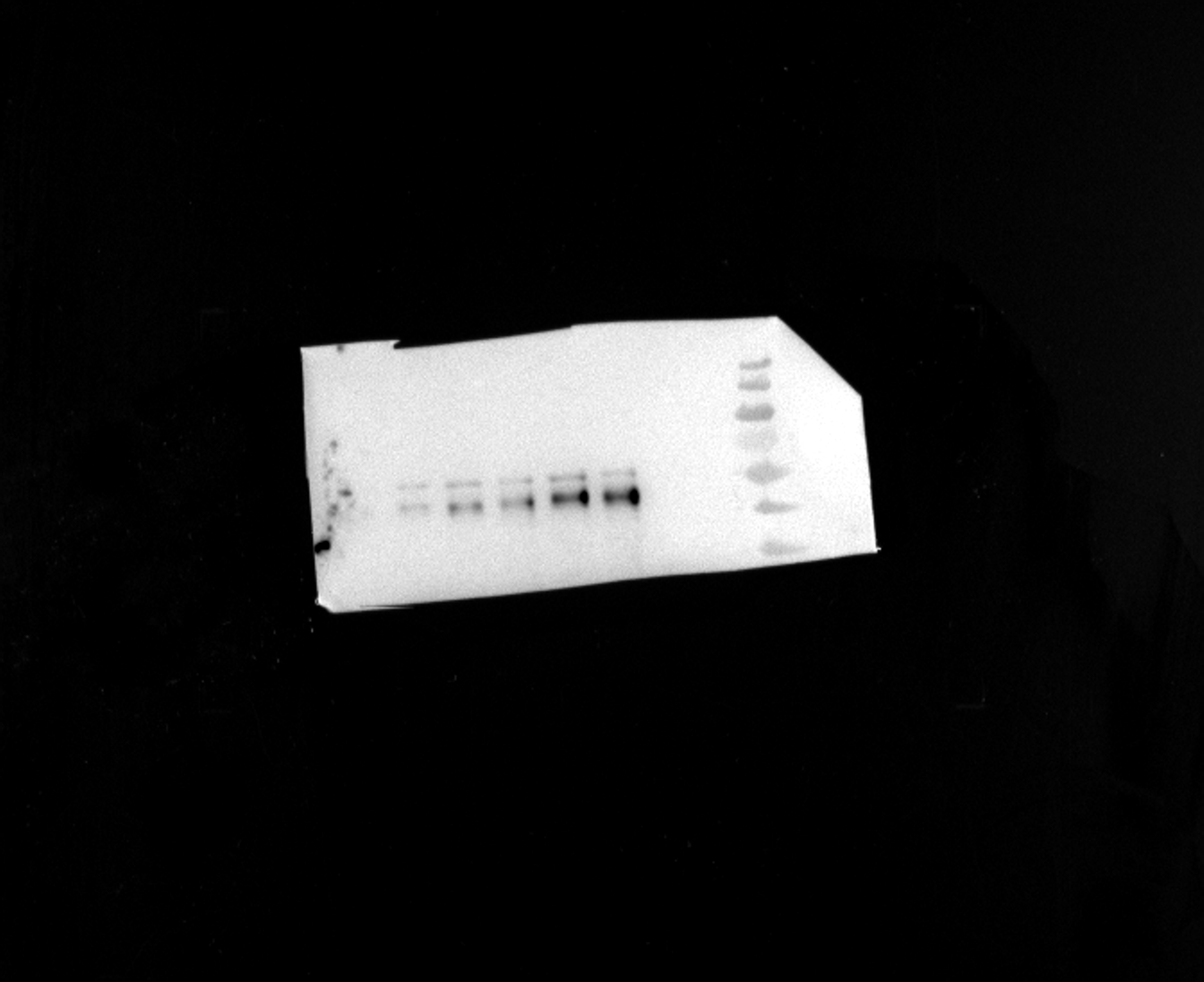

Supplement: Supplemental Information 6 [file peerj-10-13381-s006.tif]

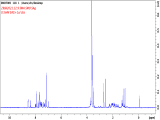

Supplement: Supplemental Information 8 [file peerj-10-13381-s008.zip › 118/pdata/1/thumb.png]

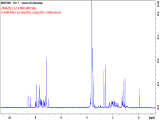

Supplement: Supplemental Information 8 [file peerj-10-13381-s008.zip › 119/pdata/1/thumb.png]

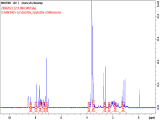

Supplement: Supplemental Information 8 [file peerj-10-13381-s008.zip › 120/pdata/1/thumb.png]

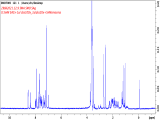

Supplement: Supplemental Information 8 [file peerj-10-13381-s008.zip › 121/pdata/1/thumb.png]

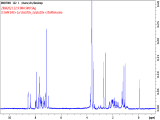

Supplement: Supplemental Information 8 [file peerj-10-13381-s008.zip › 122/pdata/1/thumb.png]

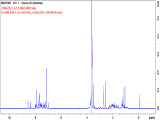

Supplement: Supplemental Information 8 [file peerj-10-13381-s008.zip › 123/pdata/1/thumb.png]

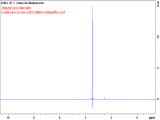

Supplement: Supplemental Information 9 [file peerj-10-13381-s009.zip › 78/pdata/1/thumb.png]

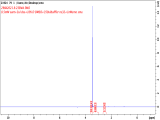

Supplement: Supplemental Information 9 [file peerj-10-13381-s009.zip › 79/pdata/1/thumb.png]

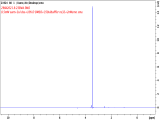

Supplement: Supplemental Information 9 [file peerj-10-13381-s009.zip › 80/pdata/1/thumb.png]

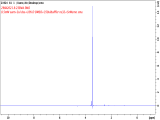

Supplement: Supplemental Information 9 [file peerj-10-13381-s009.zip › 81/pdata/1/thumb.png]

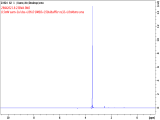

Supplement: Supplemental Information 9 [file peerj-10-13381-s009.zip › 82/pdata/1/thumb.png]

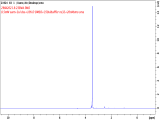

Supplement: Supplemental Information 9 [file peerj-10-13381-s009.zip › 83/pdata/1/thumb.png]

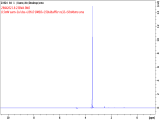

Supplement: Supplemental Information 9 [file peerj-10-13381-s009.zip › 84/pdata/1/thumb.png]
